# Trends and factors associated with early initiation of breastfeeding in Namibia: analysis of the Demographic and Health Surveys 2000–2013

**DOI:** 10.1186/s12884-018-1811-4

**Published:** 2018-05-16

**Authors:** M. N. Ndirangu, S. M. Gatimu, H. M. Mwinyi, D. C. Kibiwott

**Affiliations:** 1 Division of Social Medicine and Global Health, Department of Clinical Sciences, Lund University, Malmö, Sweden; 2 School of Nursing and Midwifery, Aga Khan University, P.O. Box 30270 – 00100, Nairobi, Kenya; 3 Kenya Red Cross Society, P.O. Box 40712 – 00100, Nairobi, Kenya; 4 School of Medicine and Health Sciences, Kabarak University, P.O. Private Bag 20157, Kabarak, Kenya

**Keywords:** Breastfeeding, Early initiation, Trends, Determinants, Namibia, Demographic health survey

## Abstract

**Background:**

Early initiation of breastfeeding (EIBF) lowers the risk for all-cause mortality in babies, including those with low birth weight. However, rates of neonatal mortality and delayed initiation of breastfeeding remain high in most low- and middle-income countries. This study aimed to assess the trends and factors associated with EIBF in Namibia from 2000 to 2013.

**Methods:**

An analysis of EIBF trends was conducted using data from three Namibia Demographic Health Surveys. The present sample included singleton children younger than 2-years from 2000 (*n* = 1655), 2006–2007 (*n* = 2152) and 2013 (*n* = 2062) surveys. Descriptive statistics were used to analyse respondents’ demographic, socioeconomic and obstetric characteristics. Factors associated with EIBF were assessed using univariate analysis and further evaluated using multivariable logistic regression analysis.

**Results:**

EIBF significantly decreased from 82.5% (confidence interval [CI]: 79.5–85.0) in 2000 to 74.9% (72.5–77.2) in 2013. Factors associated with EIBF in 2000 were urban residence (adjusted odds ratio 0.58, 95% CI: 0.36–0.93), poorer household wealth index (1.82, 1.05–3.17), lack of antenatal care (0.14, 0.03–0.81), small birth size (0.38, 0.24–0.63) and large birth size (0.51, 0.37–0.79). In 2013, factors associated with EIBF were maternal age of 15–19 years (2.28, 1.22–4.24), vaginal delivery (2.74, 1.90–3.93), married mothers (1.57, 1.16–2.14), delivery assistance from health professionals (3.67, 1.23–10.9) and birth order of fourth or above (1.52, 1.03–2.26).

**Conclusions:**

Namibia has experienced a declining trend in EIBF rates from 2000 to 2013. Factors associated with EIBF differed between 2000 and 2013. The present findings highlight the importance of continued commitment to addressing neonatal health challenges and strengthening implementation of interventions to increase EIBF in Namibia.

**Electronic supplementary material:**

The online version of this article (10.1186/s12884-018-1811-4) contains supplementary material, which is available to authorized users.

## Background

Globally, under half of the newborns are breastfed within an hour of delivery [1]. The proportion is even lower in the African region (44%); this rate is “fair” according to World Health Organization (WHO) classification of early initiation of breastfeeding (EIBF) but falls below “very good” (90–100%) [2]. The benefits of EIBF for both mother and baby are well-documented, including reduced risk of postpartum haemorrhage [3, 4], increased mother-baby bonding, increased colonisation of the baby’s enteric system by microflora [5, 6] and reduced neonatal mortality (including among low birth weight babies) [7, 8].

Despite these benefits, the rate of EIBF in most middle-income countries remains low, including in Namibia. For example, EIBF rates are 30.8 and 41.9% in rural and urban Nigeria respectively [9], 44.7% in Algeria and 58.7% in Kenya [10]. Low EIBF rates are associated with unskilled birth attendance [11], non-health facility [12] and caesarean deliveries [10, 13] and maternal complications [10]. High EIBF rates are associated with health facility delivery, large birth size, formal education, urban residence, wealthier household index, non-working mothers, higher birth order and female babies [9, 13–15].

In the early 1990s, the WHO and United Nations Children’s Fund (UNICEF) launched the Baby Mother-Friendly Initiative (BMFI) to support breastfeeding practices. Namibia adopted this initiative in 1992 and was among the first African countries to launch BMFI [16]. In Namibia, BMFI resulted in training healthcare professionals on breastfeeding management and promotion, certification of all 35 hospitals as baby-friendly [17] and an increase in EIBF from 52% in 1992 to 81% in 2000 [18]. However, in recent years, Namibia has experienced changes that have posed threats to these gains. The prevalence of human immunodeficiency virus (HIV) among pregnant women receiving antenatal care increased from 4.2% in 1992 to 19.3% in 2000, with breastfeeding contributing 30–40% of mother-to-child transmission of HIV [19]. Namibia was also reclassified as an upper middle-income country in 2009, but rates of unemployment and poverty remain high. To date, no studies in Namibia have investigated the potential effects of these changes on child health indicators, including EIBF.

Namibia also failed to meet targets for child health specified in the Millennium Development Goals (MDGs) [20]. Recent UNICEF estimates indicate the EIBF rate declined from 81% in 2000 to 71% in 2016 [1, 20]. This downward trend and the lack of evidence on changes over time in factors associated with EIBF necessitate further investigations using nationally representative data. This study aimed to assess trends and factors associated with EIBF in Namibia from 2000 to 2013.

## Methods

### Data sources and sample

This study used nationally representative child datasets from the Namibia Demographic and Health Surveys (NDHS) for 2000, 2006–2007 and 2013 [18, 20, 21]. All surveys used a two-stage stratified cluster sampling design based on administrative regions and locations [18, 20, 21]. The first stage involved identification of primary sampling units and the second involved selection of households. Both stages were based on the sampling frame used in the Namibia Population and Housing Census preceding the NDHS (1991, 2001 and 2011). Individual households were selected using systematic sampling [18, 20, 21]. A trained team of interviewers using standardised pre-tested household, women’s and men’s questionnaires (translated into six local languages) collected data for all surveys [18, 20, 21].

This study used data for households and women aged 15–49 years from the three surveys: 6849 households and 7308 women from 2000; 9970 households and 10,352 women from 2006 to 2007; and 11,004 households and 9940 women from 2013. The household response rate was 96.9% in 2000, 92.3% in 2006–2007 and 97% in 2013; individual response rates for women were 92.4, 94.7 and 92%, respectively. In the 5 years preceding each survey, 3989 (2000), 5168 (2006–2007) and 5046 (2013) participating women had given birth; 1707, 2206 and 2122 children were younger than 24 months (Additional file 1: Figure S1). The present analysis included children younger than 24 months who were singleton live births (2000, *n* = 1655; 2006–2007, *n* = 2152; 2013, *n* = 2062). Detailed information on NDHS data sources, survey settings and sampling strategies have been described elsewhere [18, 20, 21].

### Measures

#### Outcome variable

The main study outcome was EIBF, which was assessed among children younger than 24 months. EIBF is defined as putting a newborn baby to the breast within 1 h of birth [22]. The NDHS assessed EIBF by asking respondents, ‘How long after birth did you first put (last born child’s name) to the breast?’ [18, 20, 21]. Responses were categorised into those who started breastfeeding within 1-h of birth and more than 1-h after birth.

#### Explanatory variables

We reviewed previously published studies on factors associated with EIBF to identify potential confounders, which were classified as maternal, obstetric and child-related factors. Maternal factors included age (< 20 years, 20–34 years, ≥35 years) [15, 23], marital status (never married, married/cohabiting, widowed/divorced/separated), residence (urban or rural), education (no formal, primary, secondary, tertiary), occupation and household wealth [13, 15, 24, 25]. Maternal occupation was categorised as paid work (skilled/unskilled manual work, clerical, blue collar), agriculture (paid and unpaid agricultural work) and unemployed (not working, housewives, domestic work) [25]. Household wealth was categorised in quintiles (1–poorest; 5–richest) [26], generated from wealth scores calculated using principal component analysis of household assets [27].

Obstetric factors included antenatal care (ANC) visits (0, 1–3, ≥4, do not remember) [3, 28], place of delivery (health facility or home/other) [3], delivery mode (vaginal or caesarean section) [29] and assistance during delivery (health professional, traditional birth attendants [TBAs], self/relatives) [24]. Child-related factors were sex [13], birth size (small, average, large) [25] and birth order (1, 2–3, ≥4) [24].

#### Statistical analysis

Data were checked for completeness and consistency. Statistical analyses on complete cases were performed with STATA version 13.1 excluding 111 (6.7%), 209 (9.7%) and 155 (7.5%) children who had missing data on the outcome variable for 2000, 2006–2007 and 2013 surveys, respectively. We used frequencies and percentages to report sample characteristics and EIBF trends, and chi-square tests to assess associations between explanatory variables and EIBF in each survey. Simple and hierarchical multivariable logistic regression analyses were conducted to assess factors associated with EIBF in each survey. To allow comparability across the surveys, variables in all three surveys with a *p*-value > 0.25 in univariable regression analyses were excluded from the multivariable analyses [30]. Maternal, obstetric, and child-related factors were first included in the model separately and then together in a final model. We also adjusted for the sampling weight and cluster design of the surveys [31] and reported unadjusted and adjusted odds ratios (OR) with 95% confidence intervals (CI).

## Results

### Respondents’ characteristics

Table 1 outlines the children’s and mothers’ characteristics. The proportion of teenage mothers decreased from 11.4% in 2000 to 10.8% in 2013, and that of mothers living in urban areas increased from 33.2% in 2000 to 47.6% in 2013. Mothers with no education decreased from 13.3% in 2000 to 5.5% in 2013, and rates of secondary education increased from 49.1% in 2000 to 66.7% in 2013. The proportion of health facility deliveries increased from 76.9% in 2000 to 88.5% in 2013. Delivery assistance from TBAs decreased from 2.2% in 2000 to 0.5% in 2013. In addition, the proportion of mothers delivering via caesarean section increased from 12.2% in 2006–2007 to 15.6% in 2013. The proportion of male babies decreased from 50.7% in 2000 to 48.3% in 2013, and that of large-sized babies increased from 30.4% in 2000 to 38.6% in 2013.

**Table 1:** Respondents’ characteristics

	2000		2006–2007		2013	
Characteristics	n	%	n	%	n	%
Age, years						
15–19	189	11.4	252	11.5	222	10.8
20–34	1124	69.0	1508	71.6	1436	71.0
35–49	297	19.6	355	16.9	381	18.2
Residence						
Urban	604	33.2	783	40.3	910	47.6
Rural	1006	66.8	1332	59.7	1129	52.4
Marital status						
Never married	664	44.4	944	44.9	1021	51.3
Married/cohabiting	841	49.9	1080	50.6	931	44.8
Divorced/separated/widow	105	5.7	90	4.6	87	3.9
Education						
No education	268	13.3	250	10.7	155	5.5
Primary	555	35.2	626	27.9	470	22.0
Secondary	751	49.1	1164	57.1	1327	66.7
Higher education	36	2.4	75	4.3	87	5.7
Occupation						
Not working	1034	66.2	1052	49.2	1203	57.1
Agriculture	66	5.8	269	12.6	36	1.4
Paid work	503	28.1	778	38.2	798	41.4
Wealth, quintiles						
Poorest	340	23.8	422	19.8	410	21.3
Poorer	298	21.9	451	20.8	410	20.3
Average	317	19.5	392	19.1	397	19.1
Richer	318	17.3	417	21.1	408	18.7
Richest	316	17.5	418	19.3	403	20.6
ANC attendance, visits						
0	113	6.1	86	3.9	82	3.2
1–3	285	18.3	378	17.7	279	13.1
≥ 4	1031	68.2	1451	71.3	1237	62.5
Do not remember	138	7.3	140	7.1	390	21.3
Place of delivery						
Health facility	1211	76.9	1704	81.9	1761	88.5
Home/others	393	23.1	410	18.1	277	11.5
Delivery mode						
Vaginal	–	–	1891	87.8	1741	84.4
Caesarean	–	–	220	12.2	295	15.6
Parity						
1	470	29.9	666	32.0	625	31.7
2–3	630	38.3	904	43.0	860	42.7
4+	510	31.9	545	25.0	554	25.6
Assistance during labour						
Health professional	1396	90.0	1944	94.5	1896	96.2
TBAs	32	2.2	28	1.3	15	0.5
Relatives/self	136	7.8	94	4.2	84	3.3
Birth order						
1	482	30.4	683	32.6	643	32.5
2–3	622	38.0	890	42.5	845	42.1
4+	506	31.6	542	24.9	551	25.5
Baby’s sex						
Male	807	50.7	1104	52.1	990	48.3
Female	803	49.3	1011	47.9	1049	51.7
Baby’s birth size						
Large	464	30.4	739	35.7	779	38.6
Average	844	53.1	1033	48.5	886	43.3
Small	291	16.5	337	15.7	392	18.1

*ANC* antenatal care, *TBAs* traditional birth attendants

### EIBF trends in Namibia

Namibia experienced a decline in the EIBF rate between 2000 and 2013. The weighted percentage of babies who were breastfed within 1 h of birth decreased significantly from 82.5% (95% CI: 79.5–85.0) in 2000 to 74.9% (95% CI: 72.5–77.2) in 2013. However, the change from 2006–2007 (72.8, 95% CI: 70.2–75.2) to 2013 was not significant.

Table 2 illustrates EIBF rates by mother and child characteristics. In all three surveys, there were equal proportions of EIBF among male and female babies, and EIBF rates were higher among mothers who lived in urban areas, delivered vaginally and in health facilities and had four or more ANC visits. EIBF was significantly associated with birth size (2000 and 2006–2007), delivery mode (2006–2007 and 2013), and occupation and marital status (2013).

**Table 2:** Rates of early initiation of breastfeeding in Namibia (2000, 2006–2007 and 2013) by demographic and socioeconomic characteristics

Characteristics	2000			2006–2007			2013			Percentage difference		
	%	95% CI	*p*-value^a^	%	95% CI	*p-*value^a^	%	95% CI	*p*-value^a^	2000–2006^b^	2006–2013^b^	2000–2013^b^
Age, years												
15–19	8.9	7.2–10.8		7.9	6.7–9.4		9.1	7.7–10.6		−1	1.2	0.2
20–34	58.2	54.4–61.9	0.143	53.1	50.4–55.8	0.057	52.7	50.0–55.3	0.160	−5.1	−0.4	−5.5
35–49	15.4	13.3–17.8		11.7	10.1–13.6		13.2	11.4–15.2		−3.7	1.5	−2.2
Residence												
Rural	27.6	23.8–31.7	0.210	27.7	24.8–30.7	0.856	34.9	32.3–37.4	0.579	0.1	7.2*	7.3**
Urban	54.8	50.9–58.7		45.1	42.5–47.8		40.1	37.4–42.8		−9.7	−5	−14.7**
Marital status												
Never married	35.8	32.3–39.4		33.2	30.5–35.9		37.1	34.4–39.7		−2.6	3.9	1.3
Married/cohabiting	42.2	38.6–45.9	0.322	36	33.3–38.8	0.497	35.1	32.5–37.8	0.021	−6.2	−0.9	−7.1
Divorced/separated/widow	4.4	3.4–5.8		3.6	2.7–4.7		2.8	2.0–3.8		−0.8	−0.8	−1.6
Education												
No formal	11.5	8.9–14.6		7.8	6.5–9.4		4.2	3.3–5.4		−3.7	−3.6**	−7.3**
Primary	29.1	25.6–32.8	0.805	21.3	19.4–23.5	0.542	17.3	15.3–19.4	0.071	−7.8**	−4	−11.8**
Secondary	39.9	36.4–43.4		40.6	37.8–43.4		50.0	47.1–53.0		0.7	9.4	10.1**
Tertiary	2.0	1.2–3.3		3.0	2.0–4.5		3.4	2.4–4.9		1	0.4	1.4
Occupation												
Not working	54.3	50.7–57.8		36.6	33.9–39.4		44.2	41.4–47.1		−12.8**	1.7	−11.1**
Agriculture	5.2	3.3–7.9	0.114	9.4	7.8–11.1	0.933	1.1	0.7–1.8	0.023	3.3	0.3	3.6
Paid work	23.0	20.2–26.0		27.0	24.1–30.0		29.6	27.3–32.0		3.3	0.3	3.6
Wealth, quintiles												
Poorest	19.9	17.1–23.1		15.1	13.1–17.2		15.7	13.7–18.0		−4.8	0.6	−4.2
Poorer	19.0	16.2–22.2		15.9	13.9–18.1		15.8	13.7–18.1		−3.1	−0.1	−3.2
Average	16.0	13.1–19.5	0.378	13.5	11.6–15.7	0.624	15.0	12.9–17.2	0.198	−2.5	1.5	− 1
Richer	13.8	11.2–16.8		14.5	12.7–16.6		14.2	12.3–16.4		0.7	−0.3	0.4
Richest	13.8	11.1–17.0		13.9	12.2–15.8		14.3	12.3–16.6		0.1	0.4	0.5
ANC attendance, visits												
0	5.4	4.0–7.3		3.1	2.3–4.3		2.6	1.9–3.5		−2.3	−0.5	−2.8**
1–3	15.3	13.3–17.5	0.523	13.5	11.9–15.2	0.379	10.3	8.8–11.9	0.063	−1.8	−3.2	−5**
≥ 4	55.2	51.2–59.0		51.4	48.5–54.2		45.7	42.8–45.6		−3.8	−5.7**	−9.5**
Don’t remember	6.3	4.7–8.3		4.8	3.9–5.8		16.4	14.2–18.8		−1.5	11.6**	10.1**
Place of delivery												
Health facility	63.5	59.9–67.0	0.870	60.0	57.1–62.7	0.342	65.9	63.4–68.4	0.456	−3.5	5.9*	2.4
Home/others	19.0	16.8–22.3		12.9	11.2–14.8		9.0	7.6–10.6		−6.1*	−3.9	−10**
Delivery mode												
Vaginal	–	–	–	66.9	64.3–69.5	< 0.001	66.2	63.6–68.7	< 0.001		−0.7	
Caesarean	–	–	–	5.8	4.6–7.4		8.7	7.2–10.5			2.9	
Parity												
1	23.3	20.9–26.0		23.6	21.2–26.3		23.9	21.8–26.2		0.3	0.3	0.6
2–3	33.0	30.4–35.7	0.093	30.9	28.6–33.2	0.466	31.3	29.0–33.7	0.086	−2.1	0.4	−1.7
4+	26.1	23.1–29.4		18.3	16.3–20.5		19.7	17.6–22.0		−7.8	1.4	−6.4**
Delivery assistance												
Health professional	73.9	70.7–76.8		68.2	65.6–70.7		72.1	69.7–74.4	0.049	−5.7	3.9	−1.8
TBAs	2.0	1.3–3.1	0.438	1.3	0.7–2.1	0.062	0.2	0.1–0.5		−0.7	−1.1*	−1.8*
Relative/self	6.2	4.7–8.2		3.3	2.4–4.4		2.6	2.0–3.5		−2.9	−0.7	−3.6**
Birth order												
1	23.8	21.3–26.4		23.6	21.2–26.3		23.9	21.8–26.2	0.086	−0.2	0.3	0.1
2–3	32.8	30.2–35.6	0.111	30.9	28.6–33.2	0.466	31.3	28.9–33.7		−1.9	0.4	−1.5
4+	25.8	22.8–29.1		18.3	16.3–20.5		19.7	17.6–22.0		−7.5	1.4	−6.1
Baby’s sex												
Male	41.2	37.8–44.6	0.426	36.4	34.0–38.9	0.284	36.8	34.4–39.2	0.134	−4.8	0.4	−4.4
Female	41.3	38.3–44.3		36.4	33.8–39.1		38.1	35.7–40.6		−4.9	1.7	−3.2
Baby’s birth size												
Small	23.5	20.4–26.8		24.6	22.2–27.2		28.6	26.1–31.1		1.1	4	5.1
Average	47.2	43.5–50.9	0.001	37.7	35.2–40.4	0.008	33.6	31.0–36.3	0.090	−9.5**	−4.1	−13.6**
Large	11.7	9.7–14.0		10.4	8.8–12.1		12.8	11.0–14.9		−1.3	2.4	1.1

*ANC* antenatal care, *CI* confidence interval, *TBAs* traditional birth attendants

^a^*p*-value for association for each year

^b^Percentage point difference between survey years with a significance tests for difference in proportions; *p-value < 0.05; **p-value < 0.01

Overall, there was a significant decrease in the proportion of mothers who initiated breastfeeding early. The EIBF rate among urban mothers decreased significantly from 54.8% (95% CI: 50.9–58.7) in 2000 to 40.1% (95% CI: 37.4–42.8) in 2013. Similarly, the EIBF rate among married mothers decreased from 42.2% (95% CI: 38.6–45.9) in 2000 to 35.1% (95% CI: 32.5–37.8) in 2013. There was a decrease in EIBF among working mothers from 57.4% (95% CI: 54.0–60.8) in 2000 to 46.3% (95% CI: 43.5–49.2) in 2013. EIBF among mothers with a secondary education increased from 39.9% (95% CI: 36.4–43.4) in 2000 to 50% (95% CI: 47.1–53.0) in 2013 (Table 2).

### Factors associated with EIBF in Namibia

In the bivariate analysis, EIBF was significantly associated with birth order and birth size in 2000, birth size, maternal age, and delivery mode in 2006–2007 and birth size, birth order, delivery assistance by TBAs, delivery mode, ANC, occupation, wealth, education and marital status in 2013 (Table 3).

**Table 3:** Univariate and multivariable logistic regression analyses of factors associated with early initiation of breastfeeding

	2000		2006–2007		2013	
Characteristics	COR (95% CI)	AOR (95% CI)	COR (95% CI)	AOR (95% CI)	COR (95% CI)	AOR (95% CI)
Age, years						
15–19	0.91 (0.51–1.63)	1.10 (0.53–2.31)	0.87 (0.57–1.34)	1.00 (0.57–1.75)	1.56 (0.96–2.56)	2.28 (1.22–4.24)*
20–34	1.38 (0.89–2.15)	1.55 (0.91–2.62)	1.26 (0.94–1.69)	1.49 (1.07–2.08)*	1.05 (0.76–1.46)	1.30 (0.90–1.87)
35–49	1.00	1.00	1.00	1.00	1.00	1.00
Residence						
Rural	1.00	1.00	1.00	1.00	1.00	1.00
Urban	0.74 (0.46–1.19)	0.58 (0.36–0.93)*	1.03 (0.77–1.36)	0.89 (0.64–1.24)	1.07 (0.84–1.38)	0.93 (0.68–1.26)
Marital status						
Never married	1.00	1.00	1.00	1.00	1.00	1.00
Married/cohabiting	1.29 (0.86–1.94)	1.23 (0.73–2.07)	0.99 (0.78–1.27)	0.94 (0.72–1.24)	1.44 (1.10–1.90)*	1.57 (1.16–2.14)*
Divorced/separated	0.93 (0.47–1.81)	0.96 (0.47–1.96)	1.44 (0.78–2.64)	1.41 (0.75–2.65)	1.09 (0.59–2.01)	1.11 (0.60–2.03)
Education						
Tertiary	1.00	1.00	1.00	1.00	1.00	1.00
Secondary	0.96 (0.38–2.47)	0.90 (0.32–2.51)	1.11 (0.55–2.26)	0.86 (0.37–1.99)	1.75 (0.98–3.11)	1.22 (0.51–2.19)
Primary	0.98 (0.41–2.39)	0.89 (0.32–2.43)	1.35 (0.66–2.76)	1.03 (0.43–2.48)	1.95 (1.07–3.56)*	1.06 (0.51–2.19)
No formal	1.23 (0.46–3.23)	0.95 (0.31–2.88)	1.28 (0.62–2.67)	0.93 (0.37–2.38)	2.31 (1.14–4.68)*	1.21 (0.51–2.86)
Occupation						
Paid work	1.00	1.00	1.00	1.00	1.00	1.00
Agriculture	4.03 (0.98–16.5)	2.87 (0.69–11.9)	1.01 (0.71–1.43)	0.91 (0.62–1.35)	1.62 (0.62–4.24)	1.55 (0.58–4.11)
Not working	1.03 (0.66–1.56)	1.05 (0.69–1.60)	1.04 (0.77–1.41)	0.98 (0.72–1.34)	1.36 (1.07–174)*	1.27 (0.97–1.66)
Wealth						
Richest	1.00	1.00	1.00	1.00	1.00	1.00
Richer	0.87 (0.56–1.35)	0.91 (0.54–1.51)	0.90 (0.63–1.29)	0.87 (0.60–1.26)	1.51 (1.04–2.02)*	1.35 (0.90–2.03)
Average	1.30 (0.81–2.10)	1.52 (0.86–2.68)	0.94 (0.68–1.30)	0.93 (0.65–1.33)	1.47 (0.98–2.20)	1.22 (0.78–1.91)
Poorer	1.36 (0.82–2.22)	1.82 (1.05–3.17)*	1.14 (0.79–1.64)	1.07 (0.73–1.58)	1.33 (0.93–1.92)	1.02 (0.67–1.56)
Poorest	1.13 (0.70–1.81)	1.52 (0.89–2.57)	1.13 (0.76–1.68)	1.08 (0.70–1.66)	1.26 (0.86–1.85)	0.94 (0.59–1.48)
ANC attendance, visits						
0	1.00	1.00	1.00	1.00	1.00	1.00
1–3	0.69 (0.28–1.70)	0.19 (0.04–1.05)	0.81 (0.44–1.52)	1.02 (0.12–8.55)	0.62 (0.30–1.26)	0.17 (0.01–3.16)
4+	0.57 (0.25–1.29)	0.14 (0.03–0.81)*	0.67 (0.37–1.20)	0.85 (0.10–7.09)	0.45 (0.24–0.84)*	0.14 (0.01–2.51)
Do not remember	0.67 (0.22–2.05)	0.18 (0.03–1.21)	0.65 (0.34–1.28)	0.79 (0.09–6.65)	0.57 (0.29–1.14)	0.16 (0.01–3.09)
Place of delivery						
Home/others	1.00	1.00	1.00	1.00	1.00	1.00
Health facility	1.03 (0.70–1.53)	1.33 (0.86–2.07)	1.13 (0.87–1.47)	1.53 (1.10–2.14)*	0.88 (0.63–1.23)	1.25 (0.83–1.90)
Delivery mode^a^						
Caesarean	–	–	1.00	1.00	1.00	1.00
Vaginal	–	–	2.53 (1.75–3.68)**	2.58 (1.68–3.97)**	2.84 (2.03–3.97)**	2.74 (1.90–3.93)**
Delivery assistance						
TBAs	1.00	1.00	1.00	1.00	1.00	1.00
Health professional	0.47 (0.15–1.47)	0.48 (0.14–1.61)	0.32 (0.10–1.01)	0.19 (0.04–0.91)*	3.60 (1.09–11.8)*	3.67 (1.23–10.9)*
Relative/self	0.44 (0.13–1.47)	0.13 (0.02–0.81)	0.47 (0.13–1.71)	0.30 (0.02–4.30)	5.31 (1.36–20.7)*	0.99 (0.05–20.7)
Birth order						
1	1.00	1.00	1.00	1.00	1.00	1.00
2–3	1.50 (1.01–2.23)*	1.36 (0.87–2.12)	1.13 (0.85–1.50)	0.99 (0.69–1.43)	1.08 (0.83–1.40)	1.16 (0.86–1.56)
4+	1.10 (0.74–1.64)	1.20 (0.67–2.15)	1.21 (0.86–1.72)	1.23 (0.78–1.95)	1.42 (1.03–1.97)*	1.52 (1.03–2.26)*
Baby’s birth size						
Average	1.00	1.00	1.00	1.00	1.00	1.00
Small	0.39 (0.24–0.63)**	0.38 (0.24–0.60)**	0.64 (0.44–0.93)*	0.63 (0.44–0.90)*	0.71 (0.51–0.99)*	0.72 (0.65–1.01)
Large	0.55 (0.37–0.79)*	0.51 (0.34–0.78)*	0.67 (0.51–0.90)*	0.69 (0.51–0.94)*	0.83 (0.64–1.07)	0.86 (0.52–1.13)

^a^Delivery mode was not available for the 2000 NDHS. However, this was measured in the later surveys

*AOR* adjusted odds ratio, *CI* confidence interval, *COR* crude odds ratio (unadjusted odds ratio), *NDHS* Namibia Demographic Health Survey, *TBAs* traditional birth attendants **p-value < 0.001 *p-value < 0.05

The multivariate analysis showed that in 2000, the odds of EIBF were 82% higher among mothers in households with a poorer wealth index compared with richer households (AOR 1.82, 95% CI: 1.05–3.17). In addition, mothers in rural areas had 42% (AOR 0.58, 95% CI 0.36–0.93) reduced odds of EIBF compared with urban mothers. Mothers who attended the recommended four or more ANC visits had 86% (AOR 0.14, 95% CI: 0.03–0.81) reduced odds of EIBF compared with those not attending ANC. In 2006–2007, the odds of EIBF were 49% higher among mothers aged 20–34 years compared with those aged ≥35 years, 53% higher among health facility deliveries compared with home deliveries and 2.58 times higher among those who had a vaginal delivery compared with a caesarean section. In 2013, mothers aged 15–19 years (AOR 2.28, 95% CI: 1.22–4.24), married mothers (AOR 1.57, 95% CI: 1.16–2.14) and those who had a vaginal delivery (AOR 2.74, 95% CI: 1.90–3.93) had higher odds of EIBF compared with older women, unmarried women and those who had a caesarean section, respectively. Babies with a birth order of fourth or above had 52% increased odds of EIBF compared with first-born babies (AOR 1.52, 95% CI: 1.03–2.26). In 2006–2007, health professional-assisted deliveries had 81% reduced odds of EIBF compared with TBAs-assisted delivery (AOR 0.19, 95% CI: 0.04–0.91). In contrast, the odds of EIBF among health professional-assisted deliveries in 2013 were 3.67 times higher compared with TBAs-assisted deliveries (AOR 3.67, 95% CI: 1.23–10.9) (Table 3).

## Discussion

The WHO classifies EIBF rates as poor (0–29%), fair (30–49%), good (50–89%) and very good (90–100%) [2]. The EIBF rate in Namibia is still considered good despite the significant decline from 82.5% in 2000 to 74.9% in 2013. This trend is similar to those witnessed in other middle-income countries such as Vietnam (62%) and Haiti (69%) [29, 32], and in lower-middle income settings (82%) [33–35]. The decline in the EIBF rate in Namibia between 2000 and 2006–2007 may be attributed to the high rates of HIV [19], insufficient health infrastructure, poor access, and ineffective and inefficient health service provision [36]. However, the slight increase in the EIBF rate between the 2006–2007 and 2013 surveys may be explained by government efforts, such as enactment of infant and young child feeding policies [36].

Our findings showed the number and nature of factors associated with EIBF varied from 2000 to 2013. Overall, this may be attributable to sociocultural and economic changes, increased rural-urban migration, improved school enrolment among girls, reduced teenage pregnancies, increased employment among women and health service use [37]. We found significant associations between EIBF and delivery mode, ANC attendance, baby’s birth size, place of residence, maternal age, marital status, delivery assistance and birth order. EIBF was also more likely among mothers who had a vaginal delivery compared with a caesarean section [13, 33, 38–40]. This may be partly explained by the increased rates of caesarean sections in 2006–2007 and 2013, effects of anaesthesia delaying the onset of lactation, associated respiratory distress among babies delivered by caesarean section [15] and healthcare professionals’ increased preoccupation with assisting mothers to stabilise rather than initiating breastfeeding [11, 41]. This highlights the need for appropriate guidelines to reduce the number of caesarean deliveries and educate mothers about the negative association between pre-labour caesarean delivery decisions and implications for the baby’s wellbeing.

Lower ANC attendance leading to delayed initiation of breastfeeding in 2000 was consistent with previous studies [33, 42]. It is paramount to promote skilled birth attendance and baby-friendly initiatives in health facilities [22] and improve new mothers’ breastfeeding practices through nutrition education during ANC visits [43]. Findings from Nigeria and Brazil indicate EIBF was more likely among mothers who had large babies at birth [9, 14, 25, 26]. In contrast, we found that EIBF was less likely among both large- and small-sized babies between 2000 and 2006–2007 compared with average-sized babies. Small babies often have weak breastfeeding reflexes, poor coordination, and difficulty swallowing [14, 26]. This may be attributable to healthcare providers’ increased attention to stabilising the baby rather than easing the initiation of breastfeeding [26]. Existing literature also shows that both mothers and healthcare providers perceive large babies as healthy, leading to EIBF [26].

We found EIBF was more likely among urban mothers compared with rural mothers, which was consistent with previous studies [14, 44]. This may be explained by higher ANC attendance rates, increased levels of employment and higher education levels among mothers living in urban areas. Urban women may also have increased access to information, leading to higher EIBF rates [45]. However, in 2013, place of residence was no longer a significant factor. This may be because of increased urbanisation and service provision to various parts of the country [20].

Younger women and adolescents had increased odds of initiating breastfeeding within the one-hour post-delivery period, which was similar to findings from low and middle-income countries [10, 46]. This may be attributable to improved girls education, numbers of planned pregnancies and social support [32], and the intention to breastfeed and better prenatal attitude [47, 48]. Moreover, maternal age as a determinant of EIBF is largely dependent on the presence of factors such education level and residency; in the absence of those factors, age may not impact the EIBF rate [10]. Other factors associated with EIBF included marital status and birth order. EIBF was more likely among married mothers and babies born into large families, which may be because of psychosocial support from family [49]. Multiparous women also have an increased level of knowledge and experience, and EIBF may, therefore, be more likely.

We did not find a significant association between socioeconomic status and EIBF, which was consistent with a study on trends and determinants of EIBF in Vietnam [29, 44]. Education, occupation, and wealth were not significantly associated with EIBF, except in 2000 where women in the poorer quintile had increased odds of EIBF. This finding may show the diminishing influence of socioeconomic factors on the uptake of health services and health information in Namibia. However, our finding differs from reports from Ethiopia [13] and Ireland [50] that showed EIBF was more likely among employed women, and from Indonesia [12] where it was less likely among women from wealthy households.

### Strengths and limitations

The use of publicly available, nationally representative data in this study allows our findings to be generalised to Namibia. However, our study has some limitations and caution is needed in interpreting the results. First, data collection for our main outcome relied on respondents’ recall, meaning there is a likelihood of recall bias. Existing literature shows that overestimation or underestimation of EIBF is possible because of the mothers’ inability to assess time in minutes or hours [51]. Moreover, it has been found that a mother’s response to the question on when they first put their baby to the breast ‘was related to the first time the newborn received breast milk rather than their first attempt to initiate breastfeeding’ [51]. Second, data on delivery mode were not available before 2006–2007. Thus, we could not assess the trends and association of delivery mode and EIBF in 2000, but the later years showed a consistent pattern. Lastly, causality cannot be inferred as this was a cross-sectional study.

### Policy and practice implications

UNICEF and WHO are implementing a global initiative to improve breastfeeding outcomes with a goal of improving the average EIBF rate to 70% globally [1]. Namibia has achieved this target because of government commitment through the implementation of policies and programmes (e.g. Food and Nutrition Policy for Namibia, National Policy on Infant and Young Child Feeding, Food and Nutrition Guidelines) and a focus on accelerating the achievement of better child health indicators since 1993 [52]. However, the EIBF rate has declined over the recent years, eroding the gains of various programmes and policies. There is a need for increased focus on reviewing existing breastfeeding policies and ensuring full implementation of relevant breastfeeding policies and programmes such as BMFI to accelerate progress towards reversing this trend of declining EIBF in Namibia and contribute to achieving the sustainable development goals 3 on health.

## Conclusion

Namibia experienced a declining trend in the EIBF rate from 2000 to 2013. Factors associated with EIBF also changed over the years. In 2000, urban residence, poorer women, ANC attendance and baby’s birth size were associated with EIBF. Associated factors in 2013 were maternal age, marital status, caesarean section, TBA-assisted delivery, and birth order. These findings suggest there is a need for renewed commitment to promote breastfeeding in Namibia to reverse the trend of declining EIBF.

## Additional file

Additional file 1: **Figure S1.** Flow diagram showing how the sample was obtained, provides information on the survey years, the number of households surveyed and the response rate both at the household level and for the women, detailing the number of births in the preceding 5 years, babies aged < 24 months old and the singleton babies. (DOCX 69 kb)

## Abbreviations

ANC: Antenatal care
AOR: Adjusted odds ratio
BMFI: Baby Mother-Friendly Initiative
CI: Confidence interval
COR: Crude odds ratio
EIBF: Early initiation of breastfeeding
HIV: Human immunodeficiency virus
MDGs: Millennium development goals
NDHS: Namibia Demographic Health Survey
OR: Odds ratio
TBAs: Traditional birth attendants
UNICEF: United Nations Children’s Fund
WHO: World Health Organisation
