# Supplementary figures and images for: Trends and factors associated with early initiation of breastfeeding in Namibia: analysis of the Demographic and Health Surveys 2000–2013
Source: BMC Pregnancy Childbirth. 2018 May 16;18:171. doi: 10.1186/s12884-018-1811-4 (PMC5956738; doi:10.1186/s12884-018-1811-4)

**Additional File 1 – Figure 1: Flow diagram showing how the final sample was obtained**


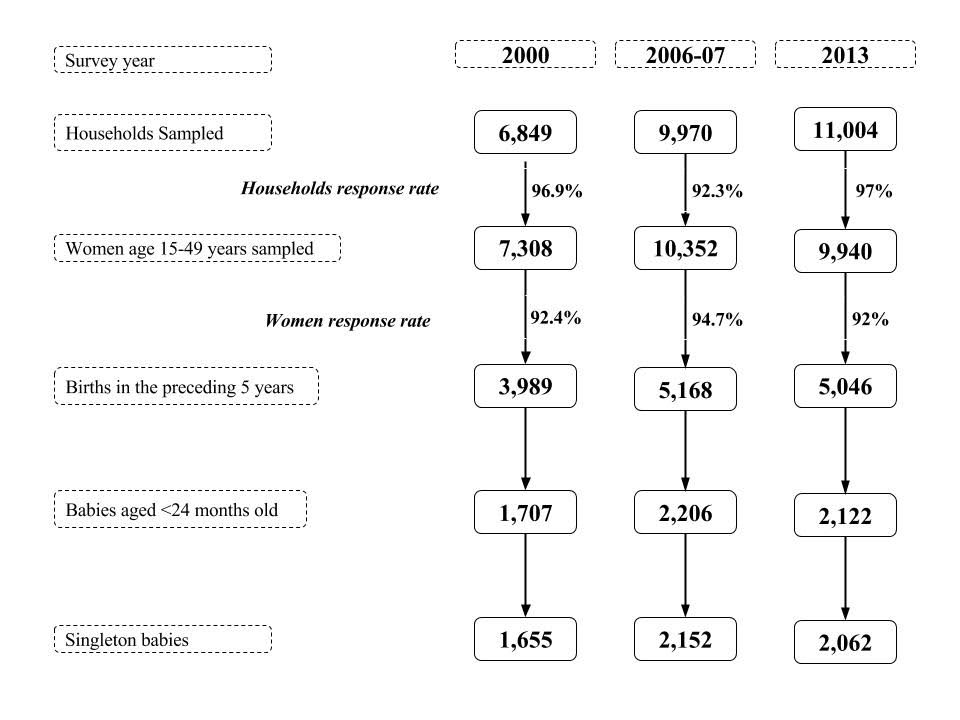

Supplement: Supplementary file 1 — Figure S1. Flow diagram showing how the sample was obtained, provides information on the survey years, the number of households surveyed and the response rate both at the household level and for the women, detailing the number of births in the preceding 5 years, babies aged < 24 months old and the singleton babies. (DOCX 69 kb) [file 12884_2018_1811_MOESM1_ESM.docx]
